# The role of pharmacists in supporting service users to optimise antipsychotic medication

**DOI:** 10.1007/s11096-023-01630-9

**Published:** 2023-09-13

**Authors:** Jo Howe, Laura Lindsey

**Affiliations:** 1 School of Pharmacy, Aston University, Birmingham, B4 7ET England; 2 Faculty of Medical Sciences, School of Pharmacy, Newcastle University, Newcastle upon Tyne, NE1 7RU England

**Keywords:** Antipsychotic medication, Medicines optimisation, Serious mental illness, Shared decision-making

## Abstract

Pharmacists have a contribution to make in improving optimising medication use for people on antipsychotic medication. The rates of prescribing antipsychotics have increased in England with an 18% rise from 2015 to 2020. People on antipsychotic medication are not treated as equal partners in conversations about their medications. This can leave people to make decisions about their antipsychotic medications without input from their prescribers which can have significant consequences for individuals. Involving people in the decision-making process, as experts on their own condition, has the potential to improve treatment outcomes. The evidence suggests that involving pharmacists in supporting people with serious mental illnesses will lead to improved clinical outcomes. Key areas for pharmacist involvement are providing information, education and counselling on antipsychotic medication and the side effects and reducing polypharmacy especially when antipsychotics are prescribed off license.

## Background

### Medication management of severe mental illness

Antipsychotic medication is the main treatment option for managing severe mental illness (SMI) both in the acute illness phase and for longer-term management [1]. Maintenance doses of antipsychotic medication are associated with lower relapse rates [2]. Although a meta-analysis of randomised controlled trials has determined the efficacy of antipsychotic medication in controlling SMI symptoms [3], they can cause debilitating side effects such as tardive dyskinesia, reduced motivation, sexual dysfunction, and cognitive impairments [4, 5]. The life expectancy of people with SMI is approximately 15–20 years less than the general population due to medication related co-morbidities, such as type II diabetes, cardiovascular disease, and high suicide rates [6, 7]. Pharmacists can make significant contributions to the optimisation of medication for individuals prescribed antipsychotics.

Internationally, prescribing rates for antipsychotics have risen [8]. In England, from 2015 to 2020 an 18% increase in prescriptions of antipsychotic medication was reported [9]. Approximately half these prescriptions were due to schizophrenia or bipolar disorder diagnosis [10]. This increase has been attributed to off label prescriptions [11]. Despite robust evidence to determine efficacy, antipsychotic medication is routinely used off label for conditions like insomnia, eating disorders and post-traumatic stress as well as reducing agitation in older patients with dementia [12, 13]. Rates of prescribing antipsychotics to children and adolescents has also grown for conditions like autism spectrum disorders and attention deficit hyperactivity disorder [14, 15]. They are often used for their sedative properties by controlling behaviour, but their effectiveness over non-pharmacological treatments has not been proven [15, 16]. Given the seriousness of the side effects and the risks of developing serious physical health conditions, or mortality [17, 18], the appropriateness of antipsychotics with these populations should be questioned.

Individuals taking antipsychotics should be regularly monitored and involved in decisions about their care. Yet, care for mental health is often episodic rather than a continuum. Once someone is stable and psychotic symptoms are in remission, on-going monitoring focuses on physical health risks such as cardiac and metabolic health [19], However, physical health monitoring is challenging and improvement of system–related factors is needed[20]. Service users and carers feel unsupported, and GPs are hesitant to advise on antipsychotic medication [21], furthermore, access to specialist services is at the point of acute need [22].

### Living with antipsychotic medication

There is a paucity of research focussing on service users’ perspectives [23]. Qualitative research has found some service users appreciate reductions in symptoms, whereas others believe medication has harmed them [2, 24, 25]. Consequently, adherence is problematic, with rates varying widely [26, 27]. Practitioners and researchers on adherence problems in SMI believe lack of service user insight is the most significant reason influencing medication adherence [28], whereas service users cite debilitating side effects of antipsychotic medications as significant obstacles to adherence [29], with estimates that nearly 75% of service users discontinue medication within 18 months due to perceived inefficacy and intolerable side effects [30].

These discrepancies in reasons for non-adherence can be somewhat explained by understanding that practitioners can underestimate the impact taking antipsychotic medication has on quality of life [31]. For service users, adherence is a balancing act to determine if reductions in psychotic symptoms outweigh potentially debilitating side effects [25]. Quality of life has been linked with reduced relapse rates [32–34]. Adherence, however, is not simply service users complying with prescribed medication but involves gaining an understanding about the needs and preferences of service users, and in the context of shared decision-making, help them make choices about medication and treatment options.

### Shared decision-making as a tool for empowerment

Due to the impact of antipsychotic medication side effects on quality of life, service users may wish to discuss, reducing or discontinuing antipsychotics [25]. However, true shared decision-making is difficult to achieve. service users often feel dismissed or not believed [21, 35, 36], which has been partially attributed to practitioners perceptions that service users are unable to participate in shared decision-making due to lack of insight [37]. Some practitioners limit or withhold information [38] believing disclosure of side effects will negatively influence adherence, and result in relapse [39, 40]. Also, practitioners often wait for service users to initiate conversations about side effects [41].

Even with well-controlled SMI symptoms, practitioners can be reluctant to fully engage in shared decision-making with service users wanting to discuss changing medication regimes [22, 42]. This reluctance can be sensed; some service users then take control of their medication decisions, without the knowledge of their practitioners [24, 25]. service users are often unaware of withdrawal effects or the need to taper [43], abrupt discontinuation can increase relapse rates [44, 45] or withdrawal symptoms leading to unintended harm [13]. This strongly suggests service users are not empowered to make informed choices about their medication.

### Facilitating medication optimisation through information

Adherence to antipsychotic medication is difficult to evaluate as it generally relies on self-reported declarations. Research has indicated adherence may be improved if service users are knowledgeable about side effects as this can lead to reductions in stress and help service users be better prepared, more in control, and able to cope with the side effects [38, 46]. Service users wish to be involved in decisions about their health but often feel they receive insufficient information or it is in a format which is difficult to interpret [47, 48]. Information delivered via a trusting therapeutic alliance, in a tone and language easily understood by service users has better outcomes [37, 49]. Information should flow in both directions [46, 50] as practitioners need to obtain accurate pictures of service users functioning to appropriately prescribe medication. Ensuring continuity with practitioners is important for effective two-way communication as trusting therapeutic relationships develop over time [51, 52]. A lack of trust can rise from previous negative experiences or sectioning under the Mental Health Act, the main piece of legislation covering the assessment, treatment and rights of people with mental health diagnoses in the UK [53]. Therefore, practitioners need to be cognisant of service users potential previous negative encounters and willing to invest heavily in re-establishing trustful relationships.

### Challenges of implementing shared decision-making

Shared decision-making within SMI is fraught with complexity. Enforced treatment under the Mental Health Act may be appropriate as service users can lack capacity for informed decisions [54]. Psychiatrists have expressed support for shared decision-making, however, they believe service user lack of insight is a barrier [55]. Service users are more likely to disagree or resist decisions they were not involved in making [56]. service user time with prescribing practitioners, typically psychiatrists, is limited [57]; service users often require more information about diagnoses and medications [58] and seek this from other sources e.g., peers with lived experience [57], the internet (including information websites and social media chatrooms) [42, 58] and local libraries [59]. The information gained gives service users more knowledge, increases the control they have over decisions affecting their care, and can positively influence how they view medication [48, 57]. A concern, however, is that the information may be inaccurate.

### The role for pharmacists in medication optimisation

Service users need to be able to approach trusted healthcare professionals to clarify and verify accuracy of information. Pharmacists being more accessible than psychiatrists, are ideally placed to provide support and advice to service users outside of appointments with prescribing practitioners. However, service users are largely unaware of what pharmacists can offer them [60–62].

The potential for involving pharmacists in medication related conversations and prescribing for SMI has been acknowledged but how this is best implemented has not been established [60]. Qualitative research has demonstrated that mental health pharmacists’ believe shared decision-making is linked to positive clinical outcomes such as increased adherence, better therapeutic relationships, and service user satisfaction [61]. Involvement of pharmacists with mental health expertise as part of the multidisciplinary team may improve medication-related outcomes such as effectiveness and adherence [63]. In Hospital-in-the-Home care model implemented within a Mental Health Hospital, an integrated clinical pharmacist within the multidisciplinary team resulted in increased medication safety [64]. Interventions involving pharmacists aimed at supporting service users with SMI have resulted in better clinical outcomes as well as better service user reported outcomes [65].

Although pharmacist involvement in educating and counselling service users with SMIs has been associated with positive impacts on clinical outcomes [65], there are implementation challenges. The current structure of healthcare delivery means a lack of established processes results in pharmacists being unable to offer the full scope of clinical support they are capable of, to both service users and their healthcare colleagues, as they are not fully integrated within community healthcare teams [66]. Also many interventions involving pharmacists have been too complex in their design to establish which component has the greatest impact [65].

The lack of understanding within the wider healthcare profession about SMI, which includes its manifestations, embarrassment from service users about their illness, available treatment options and side effects from antipsychotic medication perpetuate societal and internalised stigma and can deter individuals living with SMI from seeking help and assistance [67]. Service users also recognise a power imbalance in shared decision-making [56, 68], and have reported barriers engaging in conversations with healthcare professionals about antipsychotic medication [68]. This leaves many in a position where treatment is not optimised, shared decisions are not made, and service users may discontinue medication without support, making them more vulnerable to withdrawal symptoms or relapse.

### Antipsychotic polypharmacy

Another area where pharmacists can make a significant contribution is in managing antipsychotic polypharmacy. Although antipsychotic polypharmacy is widespread in clinical practice, particularly for treatment resistant schizophrenia, high quality research evidence is lacking and the existing literature is mixed [43]. antipsychotic polypharmacy use has been associated with increases in mortality rates [57], unplanned hospitalisations, [69] and more complex side effect profiles. However, combining aripiprazole and clozapine was shown to reduce hospitalisations in one study [70]. Antipsychotic polypharmacy use is only recommended after all antipsychotic monotherapy and other evidence-based treatment options have been explored [43]. Given the widespread clinical use of antipsychotic polypharmacy, and the increased risk of co-morbidities e.g., diabetes and cardiovascular disease which increase with antipsychotic polypharmacy, pharmacists, through their expertise in medicines, have a key role to play in ensuring safer us of medicines.

Pharmacists can also contribute to the management of polypharmacy when antipsychotic medications are prescribed for off-label indications [71, 72]. Prescribing antipsychotics for people with dementia, even in the short-term can increase risk of adverse events and death [72] and is an important area to address [11].

### Future challenges

For the existing pharmacy workforce, knowledge and confidence about treatments for SMI have been identified as barriers for involvement in supporting service users [60, 73–75]. With the change in the UK for newly qualified pharmacists becoming prescribers at registration from 2026, additional training and exposure to people with SMIs is needed to ensure newly qualified pharmacists can appropriately support this population. Additionally, organisational barriers to pharmacist involvement in supporting service users with SMIs include lack of time and problems with information sharing between healthcare settings [71, 73].

The 3 key contributions for pharmacists, whether they are prescribers or not, in optimising antipsychotic medications for people with SMIs are:

1. supporting service users and prescribing colleagues in medication management through counselling and advice and thus improving shared decision-making.
2. improved integration within existing multidisciplinary teams, which will help improve service user outcomes and result in the wider members of the team becoming more knowledgeable about medicines.
3. focusing on reducing antipsychotic polypharmacy [71].

Successful implementation of these points are likely to be difficult; they will require a change in how the pharmacist role is conceived and integrated within healthcare settings. Pharmacists are rarely considered to be integral multidisciplinary team members, especially in community settings and this will require a change in perspective from all staff. At an organisational level, clear policy, funding, and direction can help with the integration of these valuable team members within existing multidisciplinary teams.

There is an increasing need for pharmacists to utilise their skills and expertise within existing healthcare multidisciplinary teams. Pharmacists are often considered by service users to be outside of the immediate healthcare team and therefore are uniquely placed to develop trusting relationships with service users. Furthermore, establishing ways to better involve pharmacists in encouraging effective shared decision-making in optimising antipsychotic medication and empower service users to reach appropriate and acceptable outcomes for their treatment is a priority.
